# Supplementary material for: Host Serum Amyloid A1 Facilitates Streptococcus pneumoniae Adaptation to Acidic Stress Induced by Pneumococcal Anaerobic Metabolism
Source: Microorganisms. 2025 Jun 4;13(6):1309. doi: 10.3390/microorganisms13061309 (PMC12195312; doi:10.3390/microorganisms13061309)
Supplement: Supplementary file 1 [file microorganisms-13-01309-s001.zip › microorganisms-3630004-supplementary.pdf]

Supplementary Material:

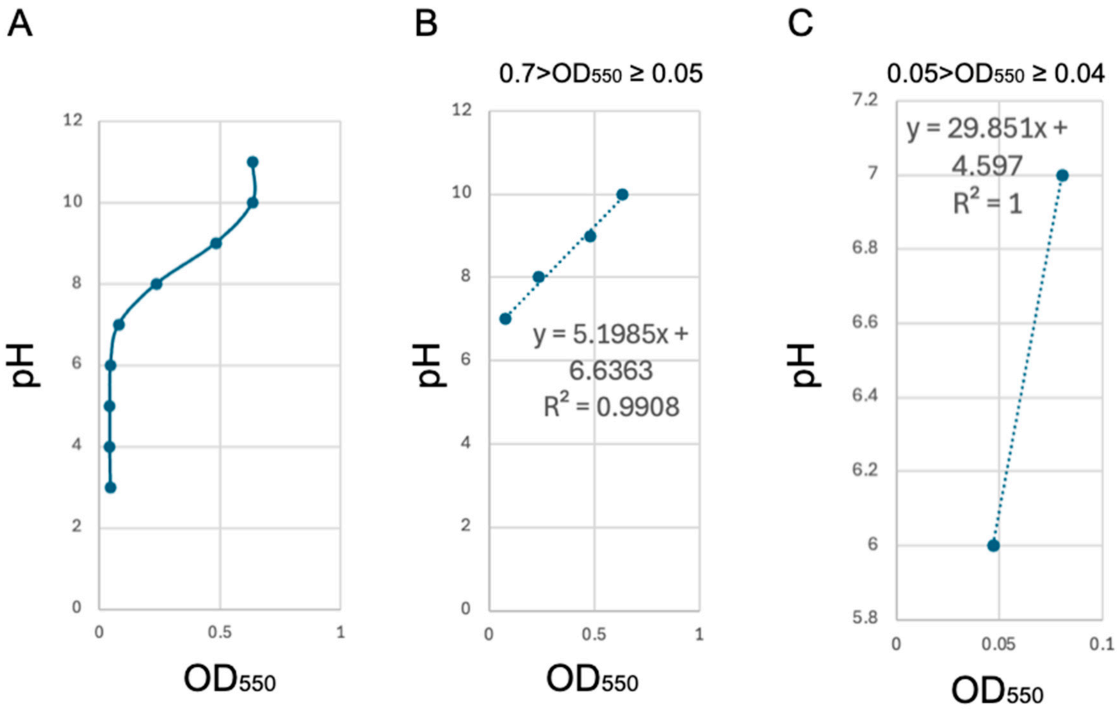

Figure S1. Estimation of pH using a calibration curve based on phenol red staining intensity. (A) A standard calibration curve was generated by adjusting the pH of THY broth from pH 3 to pH 10 using HCl or NaOH. Phenol red was added to each sample, and the absorbance at 450 nm (OD_{450}) was measured. (B) Within the linear range of OD_{450} values (0.05 to 0.7), the pH of samples can be estimated using the formula derived from the calibration curve. (C) Within the linear range of OD_{450} values (0.04 to 0.05), the pH of samples can be estimated using the formula derived from the calibration curve.

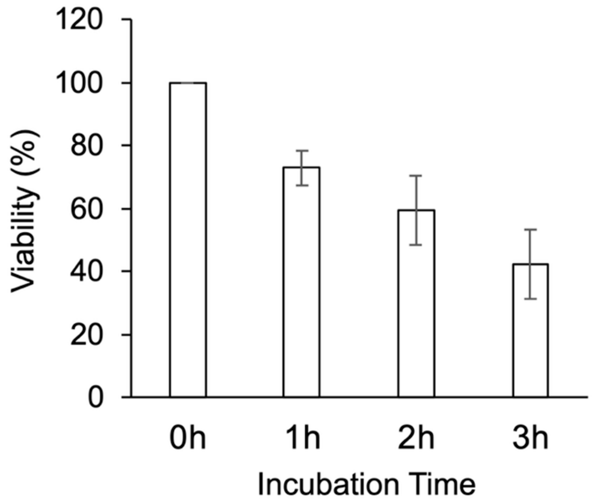

Figure S2. Survival of *S. pneumoniae* TIGR4 in 1× PBS at 37°C. TIGR4 strain of *S. pneumoniae* was suspended in 1× PBS and incubated at 37°C. At indicated time points, samples were collected, serially diluted, and plated on blood agar for CFU enumeration. The survival rate was calculated relative to the CFU count at 0 hour. Data represent the mean ± SD.

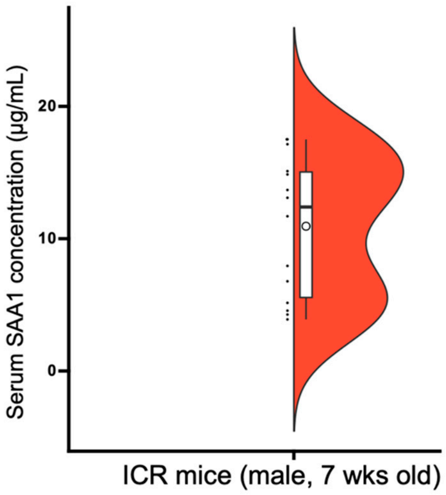

Figure S3. Serum SAA1 levels in 7-week-old male ICR mice. The concentration of SAA1 in the serum of 7-week-old male ICR mice (n = 13) was measured using SAA1 ELISA. Data represent the mean ± SD.

		PBS		THY broth	
		without 20% serum	with 20% serum	without 20% serum	with 20% serum
non-toxic	0.05% formic acid	4	4.4	6.7	7.1
lethal	0.075% formic acid	3.8	4	5.8	6.2
	0.1875% lactic acid	4	4.26	6.8	7.3
	0.125% acetic acid	4.6	5	7.1	7.4

Table. S1. pH values of PBS and THY broth supplemented with various organic acids with or without 20% serum. Formic acid, lactic acid, or acetic acid was added to either 1× PBS or THY broth to adjust the pH. The final pH of each condition was measured after the addition of the acid, either in the absence or presence of 20% (v/v) mouse serum collected from ICR mice.
